# Photoneuromodulation makes a difficult cognitive task less arduous

**DOI:** 10.1038/s41598-021-93228-2

**Published:** 2021-07-01

**Authors:** Agnes S. Chan, Tsz-lok Lee, Michael R. Hamblin, Mei-chun Cheung

**Affiliations:** 1 Department of Psychology, The Chinese University of Hong Kong, Shatin, NT, Hong Kong, China; 2 Research Center for Neuropsychological Well-Being, The Chinese University of Hong Kong, Hong Kong, China; 3 Laser Research Centre, Faculty of Health Science, University of Johannesburg, Doornfontein, 2028 South Africa; 4 Radiation Biology Research Center, Iran University of Medical Sciences, Tehran, Iran; 5 Department of Social Work, The Chinese University of Hong Kong, Hong Kong, China

**Keywords:** Psychology, Neurology

## Abstract

A positive effect of photoneuromodulation (PNM) has been found on cognitive and emotional functions in healthy populations. However, the hemodynamic changes associated with improved cognitive functions (i.e., memory and executive functions) are unexplored. Therefore, the present study investigated the hemodynamic changes associated with PNM using functional near-infrared spectroscopy (fNIRS). In this experiment, 33 young healthy adults were recruited and randomly assigned to control and experimental groups. A single PNM stimulation was applied to the forehead in the experimental group, while a sham stimulation (same procedure without machine activation) was performed for the control group. Before and after the stimulation, all participants performed an *n*-back task with 0-and 3-back conditions to assess their working memory function, and their hemodynamic responses during the tasks were measured by fNIRS. A significant group (experimental vs. control) × time (before vs. after PNM) interaction in memory-related frontal activation was found. Specifically, only the experimental group had a significant reduction in frontal hemodynamic levels during the difficult task. Additionally, the memory-related frontal activation was significantly correlated with the immediate and delayed recall of the Rey–Osterrieth Complex Figure Test assessed at baseline. Therefore, PNM may reduce the cognitive efforts needed to complete tasks with high memory loads.

## Introduction

Photobiomodulation (PBM) is a non-invasive technique that utilizes light energy with wavelengths in the visible (400–700 nm) and/or near-infrared (750–1100 nm) range to activate cellular activity. When PBM is applied to the head, it is called photoneuromodulation (PNM) and can be used to improve brain function^1^. Studies on PNM have shown positive effects on patients with dementia^2–4^, traumatic brain injury^5–8^, stroke^9–12^, and depression^13–15^. Specifically, PNM in patients with traumatic brain injury^5,6^ showed improvement in executive functioning, verbal learning, and memory after several sessions of treatment provided over several weeks to years. Additionally, a significant effect was also found in patients with dementia. By applying near-infrared PNM for 12 weeks, five patients with mild to moderately severe dementia showed an improvement in cognition^4^. Furthermore, Schiffer et al.^13^ conducted a study on ten patients with major depression and anxiety in which they received a 4-min treatment at two locations on the forehead. The results showed a reduced score on depression and anxiety scales. Cassano et al.^14^ also investigated the efficacy of PNM in patients with major depressive disorders. After receiving PNM for six sessions (2 sessions per week), 2 out of the 4 patients experienced a remission in their major depressive disorder symptoms.

The beneficial effect of PNM in improving cognitive functioning has been observed using both multiple^5,6,13,14^ and single^16–18^ sessions. To test the effect of a single session, Blanco et al.^16^ recruited 118 healthy young adults and provided a single session of 8-min PNM with a dose of 60 J/cm^2^. The results showed that the participants demonstrated a significant improvement in the learning task category. In another study, Barrett and Gonzalez-Lima^17^ provided a single session of PNM to 40 young adults, in which the participants showed improvement in sustained attention and short-term memory. In our recent study, 30 older adults performed cognitive tests assessing frontal lobe functioning (i.e., the Eriksen flanker and category fluency tests) before and after a single 7.5‐minute session of the real or sham PNM. The results showed that only the older adults who received the real PNM exhibited significant improvements in their action selection, inhibition ability, and mental flexibility after stimulation, as compared to their cognitive performance before stimulation^18^.

Although there is evidence to demonstrate the positive effects of PNM on cognitive functioning, the brain hemodynamic response behind this improvement is still not fully understood. Thus, the present study utilized functional near-infrared spectroscopy (fNIRS) to examine the hemodynamic changes in the frontal lobe after a single dose of PNM. The fNIRS, an optical imaging method that uses light in the near-infrared spectrum (650–950 nm), is a non-invasive means to monitor the hemodynamic responses evoked by brain activity^19,20^. It measures quantitative changes in the concentrations of oxygenated hemoglobin [oxy-Hb] and deoxygenated hemoglobin [deoxy-Hb] in the cerebral blood. These fNIRS signals have been shown to correlate with the blood oxygenation level-dependent signal, as measured by functional magnetic resonance imaging^21–23^ with more supply of the oxygenated blood in response to the increased brain activity in a particular region of the brain.

Causse and colleagues utilized the fNIRS to study the mental effort during cognitive tasks and found that higher [oxy-Hb] was observed when subjects are engaging in more difficult tasks than less difficult tasks^24^. The results suggested that the [oxy-Hb] can be a reliable estimate of the participants’ neural efficiency. In another study^25^, 18 older adults with mild cognitive impairment (MCI) received either a single real PNM or sham stimulation. The results showed that only those who received PNM, but not those who received the sham stimulation, demonstrated significant improvement in visual memory performance and reduced hemodynamic response as measured by fNIRS during the visual memory task. These findings suggest that PNM may reduce the cognitive efforts required to complete cognitive tasks that require high memory loads. The purpose of the present study is to examine further if similar results will be observed in young healthy adults. It is hypothesized that if an individual becomes less effortful in completing a cognitive task after receiving a single PNM session, his [oxy-Hb] level during that cognitive task will be reduced accordingly.

## Results

### Demographic and neuropsychological characteristics

The control and experimental groups were well-matched in terms of age, sex, Beck Anxiety Inventory (BAI)^26^, Beck Depression Inventory (BDI)^27^, and hours of sleep before experimentation (age, *p* = 0.18, gender, *p* = 0.85, BDI, *p* = 0.52, BAI, *p* = 0.088, subjective hours of sleep, *p* = 0.42). Concerning their visual and verbal memory abilities, both groups performed comparatively in the Hong Kong List Learning Test (HKLLT)^28^ (total learning, *p* = 0.22, 10-min delayed recall, *p* = 0.26, 30-min delayed recall, *p* = 0.23, recognition, *p* = 0.41) and Rey–Osterrieth Complex Figure Test (Rey–O)^29^ (copy, *p* = 0.38, immediate recall, *p* = 0.92 delayed recall, *p* = 0.85, recognition, *p* = 0.65). Table 1 presents the mean, SD, and the *t*/*χ*^*2*^ values.

**Table 1:** Demographics and other variables between two groups at baseline.

	Control (*n* = 15)		Experimental (*n* = 18)		*t/χ*^*2*^	*p*
	*M*	*SD*	*M*	*SD*		
**Demographics**						
Age	23.07	5.19	27.06	10.85	1.381	0.18
Gender (Female/Male)	8/7		9/9		0.036	0.85
**Mood and sleep**						
BDI	9.60	7.02	8.11	5.96	− 0.659	0.52
BAI	6.87	6.15	3.83	2.33	− 1.805	0.088
Subjective hours of sleep	7.16	1.55	6.78	1.03	− 0.826	0.42
**Memory**						
Verbal (HKLLT)						
Total Learning	34.73	5.43	32.56	4.59	− 1.249	0.22
10-min delayed recall	13.13	2.13	12.22	2.39	− 1.144	0.26
30-min delayed recall	13.40	2.32	12.39	2.38	− 1.228	0.23
Recognition	15.8	0.41	15.67	0.49	− 0.839	0.41
Visual (Rey–O)						
Copy	32.77	2.18	31.89	3.25	− 0.891	0.38
Immediate recall	19.80	6.83	20.06	7.45	0.102	0.92
Delayed recall	20.03	6.52	19.58	6.63	− 0.195	0.85
Recognition	19.93	1.94	20.28	2.27	0.463	0.65
***n*-back**						
Behavioral						
*0-back*						
A'	0.99	0.02	1.00	0.01	0.978	0.34
Reaction time	332.72	38.63	377.71	38.75	3.327	0.002
*3-back*						
A'	0.92	0.04	0.91	0.05	− 0.844	0.41
Reaction time	501.77	154.24	520.04	114.44	0.390	0.70
**fNIRS (working memory load index)**						
Overall	0.0124	0.0677	0.0974	0.1918	1.630	0.11
Left regions	0.0163	0.0795	0.0913	0.1918	1.775	0.086
Right regions	0.0085	0.0631	0.1035	0.1986	1.414	0.167

*BDI* beck depression inventory, *BAI* beck anxiety inventory, *HKLLT* Hong Kong list learning test, *Rey–O* Rey–Osterrieth complex figure test, *A'* sensitivity index.

### *n*-back related [oxy-Hb] before and after PNM

The 16 channels were divided into two groups representing the left (channels 13–16) and right (channels 1–3) in the prefrontal regions of the brain. Then, the differences in the [oxy-Hb] response between the 3-back condition and the 0-back condition were calculated as an index of the working memory load. At baseline, the [oxy-Hb] response in the left and right prefrontal regions were analyzed separately with ANOVAs of each group (control vs. experimental) and condition (0-back vs. 3-back). For the left prefrontal regions, the results showed no significant main effect on group (*F*(1,31) = 1.156, *p* = 0.29) and condition (*F*(1,31) = 4.110, *p* = 0.051), or interaction effect (*F*(1,31) = 2.000, *p* = 0.17). For the right prefrontal regions, the results showed a significant main effect on condition (*F*(1,31) = 4.384, *p* = 0.045), but the main effect on group (*F*(1,31) = 1.598, *p* = 0.22) and interaction effect (*F*(1,31) = 3.150, *p* = 0.086) were not significant.

**Figure 1:**
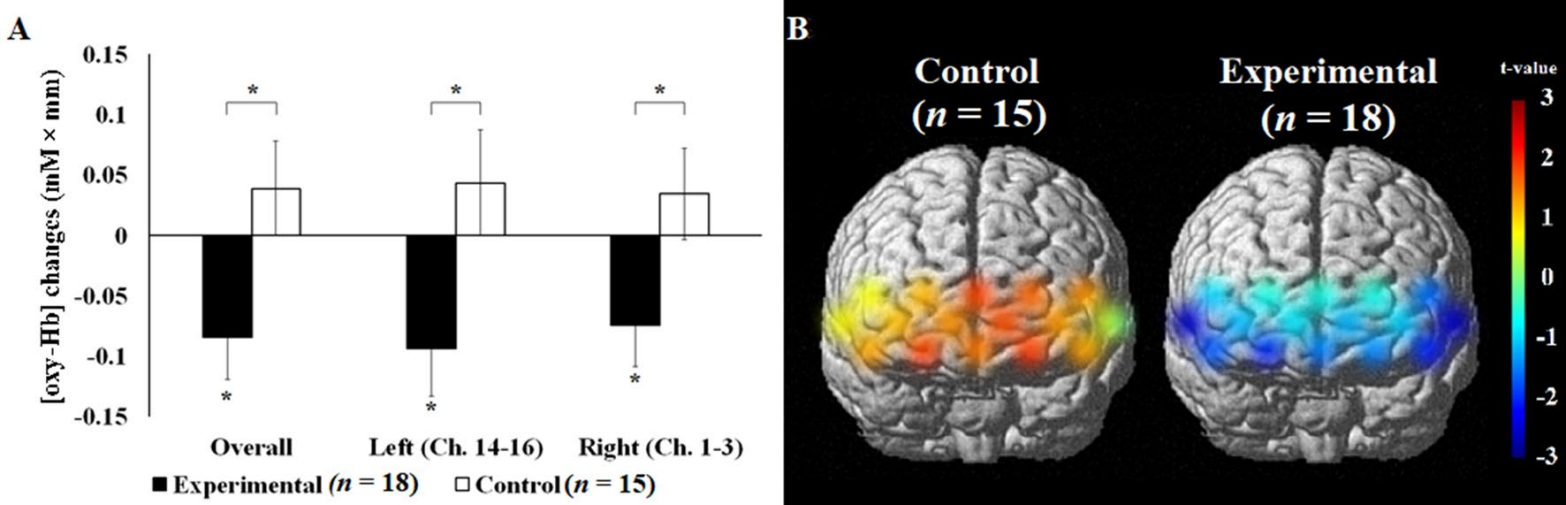
Changes in working memory-related [oxy-Hb] in the left, right, and both regions (overall) of the brain after the real/sham PNM. As seen in (**A**), after the real PNM, the bilateral prefrontal regions of the brain was less activated in the experimental group, while more activation was found in the control group. (**B**) Shows the t-statistics maps of the post-minus-pre working memory load (i.e., 3-back minus 0-back performance) in both groups. Red represents greater frontal activation related to working memory loading, while blue represents reduced frontal activation related to working memory load. The error bars represent 1 standard error of mean. * *p* < .05.

Then, the [oxy-Hb] changes between *n*-back tasks, that is, the working memory load index was evaluated after the real/sham PNM. For the control group, the working memory load index (i.e., fNIRS response of 3-back condition was subtracted from that of 0-back condition) was increased after the sham PNM, from 0.0124 to 0.0513 mM × mm; however, the difference was not significant (*t*(14) = − 0.985, *p* = 0.34). In contrast, for the experimental group, the working memory load index decreased from 0.0974 to 0.0134 mM × mm, and this difference was significant (*t*(17) = 2.403, *p* = 0.028) (Fig. 1). Given this interesting finding, the [oxy-Hb] changes before and after the real/sham PNM condition were analyzed with a repeated measures ANOVA, with before and after the real/sham PNM as the within-subject factor, and the group assignment as the between-subject factor. The multivariate result showed a significant interaction effect of the [oxy-Hb] changes in the bilateral prefrontal regions (*F*(1,31) = 5.463, *p* = 0.026, Fig. 1). The main effect of group (*F*(1,31) = 0.736, *p* = 0.40) and time (*F*(1,31) = 0.291, *p* = 0.59) in bilateral prefrontal regions was not significant.

Next, the [oxy-Hb] changes in the left and right prefrontal regions were analyzed separately. The interaction effects remained significant (left: *F*(1,31) = 5.461, *p* = 0.026; right: *F*(1,31) = 4.470, *p* = 0.043, Fig. 1). The main effects of group (left: *F*(1,31) = 0.024, *p* = 0.88; right: *F*(1,31) = 0.728, *p* = 0.40) and time (left: *F*(1,31) = 0.739, *p* = 0.40; right: *F*(1,31) = 0.599, *p* = 0.45) of the [oxy-Hb] changes in both left and right prefrontal regions were not significant.

### Relationship between working memory-related [oxy-Hb] changes after PNM, demographic variables, task performance, and working memory abilities

We then examined whether there were any factors affecting the efficacy among individuals. Pearson's correlation coefficients were calculated between the working memory-related [oxy-Hb] changes after PNM and variables on individuals' verbal and visual memory and other demographic information. There was no significant correlation between the working memory-related [oxy-Hb] changes and the demographic variables (i.e., age, BDI and BAI scores, hours of sleep) (*r* = − 0.163 – 0.432, *p* = 0.074 – 0.81).

**Figure 2:**
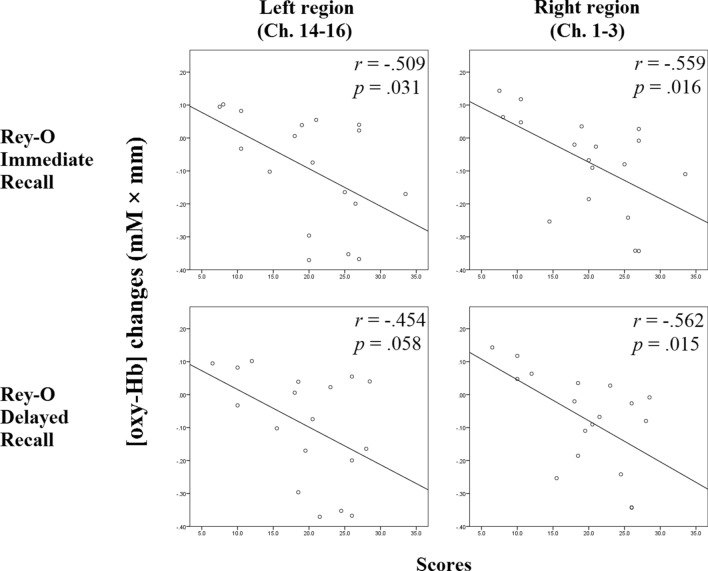
The relationship between Rey–O immediate and delayed recall and the working memory-related [oxy-Hb] changes after the real PNM. The more negative the value of the [oxy-Hb] changes (i.e., the y-axis), the more frontal deactivation was found after the real PNM.

For the visual and verbal working memory abilities, the Rey–O immediate recall correlated significantly with the working memory-related [oxy-Hb] changes in both prefrontal regions (left: *r* = − 0.509, *p* = 0.031; right: *r* = − 0.559, *p* = 0.016) (Fig. 2). Marginally significant and significant correlations were found between the working memory-related [oxy-Hb] changes and the Rey–O delayed recall (left: *r* = − 0.454, *p* = 0.058; right: *r* = − 0.562, *p* = 0.015) (Fig. 2). However, the total learning, number of unique words recalled in the 10-min and 30-min delayed recall tests, and the recognition score of the HKLLT were not significantly correlated (*r* = − 0.398 – 0.228, *p* = 0.10 – 0.95).

Regression testing was used to determine whether immediate and delayed recall in the Rey–O test could predict the working memory-related [oxy-Hb] changes after PNM. From the results, the immediate recall significantly predicted the working memory-related [oxy-Hb] changes after PNM (left: *β* = − 0.011, *t*(17) = − 2.366, *p* = 0.031; right: *β* = − 0.011, *t*(17) = − 2.697, *p* = 0.016), and explained a significant proportion of variance in the working memory-related [oxy-Hb] changes (left: *R*^2^ = 0.259, *F*(1,16) = 5.600, *p* = 0.031; right: *R*^2^ = 0.313, *F*(1,16) = 7.276, *p* = 0.016). Marginally significant and significant regressions were found for Rey–O delayed recall (left: *R*^2^ = 0.207, *F*(1,16) = 4.164, *p* = 0.058; right: *R*^2^ = 0.315, *F*(1,16) = 7.370, *p* = 0.015). The results showed that delayed recall may predict the working memory-related [oxy-Hb] changes after PNM (left: *β* = − 0.011, *t*(17) = − 2.041, *p* = 0.058; right: *β* = − 0.012, *t*(17) = − 2.715, *p* = 0.015).

### *n*-back behavioral performance before and after PNM

Before PNM, the two groups were not significantly different in terms of reaction time (*t*(31) = 0.390, *p* = 0.70) and A’ (*t*(31) = − 0.844, *p* = 0.41) in the 3-back condition. The control group responded faster than the experimental group in the 0-back condition (*t*(31) = 3.327, *p* = 0.002). However, their A’ values were not different (*t*(31) = 0.978, *p* = 0.34).

After an 8-min PNM session, for the 0-back task, the reaction time and A’ in both conditions did not change significantly in both the experimental (reaction time: *t*(17) = 1.321, *p* = 0.20; A’: *t*(17) = 0.164, *p* = 0.87) and control groups (reaction time: *t*(14) = − 0.080, *p* = 0.94; A’: *t*(14) = 0.526, *p* = 0.61). Same non-significant results were found for the 3-back task in both the experimental (reaction time: *t*(17) = 0.396, *p* = 0.70; A’: *t*(17) = 0.359, *p* = 0.72) and control groups (reaction time: *t*(14) = 1.644, *p* = 0.12; A’: *t*(14) = 1.123, *p* = 0.28). Additionally, there were significant 0-back reaction time differences between the two groups after PNM (*t*(31) = 2.540, *p* = 0.016), in which the control group responded faster than the experimental group. There were no significant differences in the A’ (*t*(31) = 1.322, *p* = 0.20) in the 0-back condition, and the reaction time (*t*(31) = 1.037, *p* = 0.31) and the A’ (*t*(31) = − 0.279, *p* = 0.78) in the 3-back condition.

**Figure 3:**
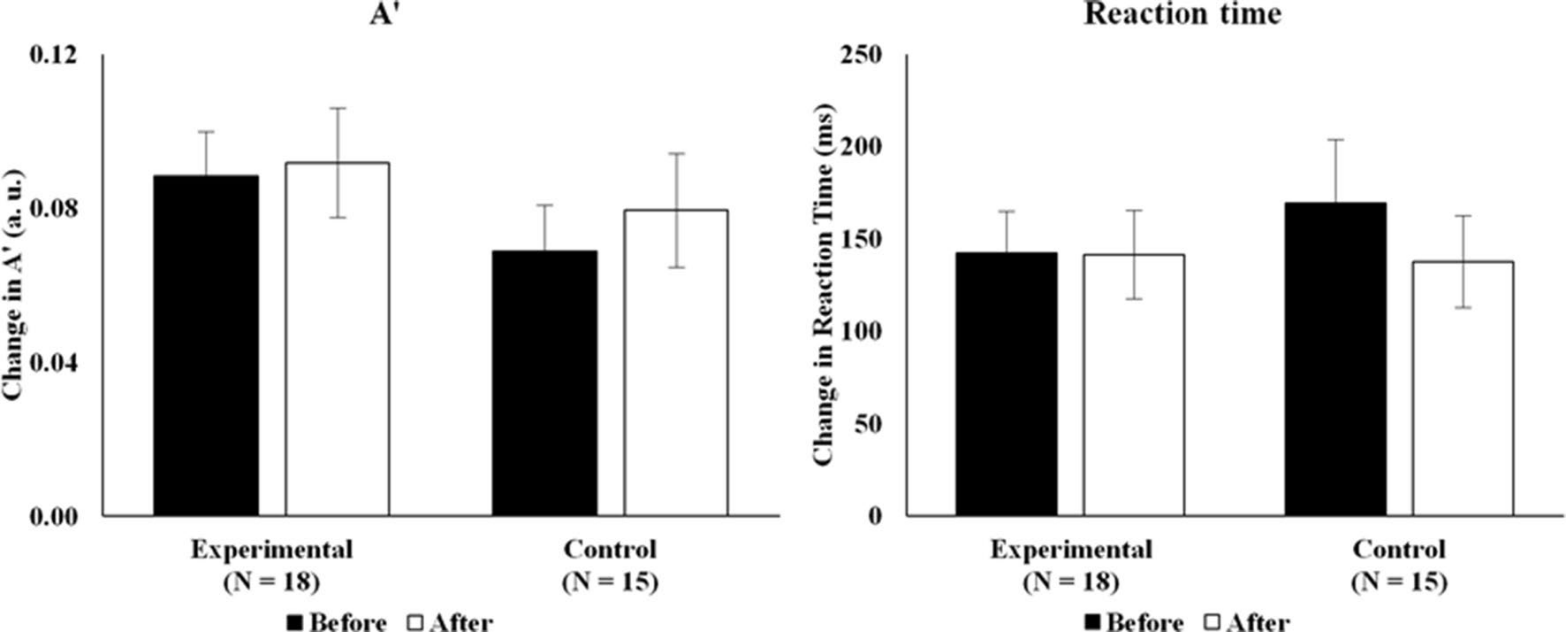
*n*-back condition difference in A' and reaction time in both groups before and after the real/sham PNM session. *Note.* A' = performance index of *n*-back task. RT = reaction time. The left graph shows the 0- minus 3-back A' difference while the right graph shows the 3- minus 0-back difference in reaction time. No significant differences within and between groups were found. The error bars represent 1 standard error of mean.

The reaction time and A’ differences between the two *n*-back conditions were calculated to investigate the participants’ working memory capacities. At baseline, there were no significant group differences in reaction time (*t*(31) = − 0.667, *p* = 0.51) and A’ difference (*t*(31) = 1.170, *p* = 0.25). Additionally, these two measures did not change significantly after the real/sham PNM in the experimental group (reaction time: *t*(17) = 0.061, *p* = 0.95; A’: *t*(17) = − 0.285, *p* = 0.78), or the control group (reaction time: *t*(14) = 1.626, *p* = 0.13; A’: *t*(14) = − 0.842, *p* = 0.41), compared to their corresponding baseline performances. Furthermore, the reaction time and A’ difference after PNM did not differ between groups (reaction time: *t*(31) = 0.107, *p* = 0.92; A’: *t*(31) = 0.591, *p* = 0.56). This means that PNM can maintain participants’ working memory abilities, indicated by the non-significance in 3- minus 0-back A’ and reaction time difference within and between the groups. Figure 3 shows the conditional difference in A’ and reaction time in both groups.

## Discussion

Previous studies have investigated the cognitive improvement after applying a single PNM session in young adults, including category learning^16^, sustained attention, memory, and mood^17^. Specifically, young adults who received a single session demonstrated a significant improvement in the learning task category^16^, and a significant improvement in sustained attention and short-term memory^17^. Similar improvements have been reported among older adults wherein they showed an improved performance on flexible thinking and inhibitory control after receiving a single stimulation^18^. The present study is consistent with previously reported studies showing significant improvement in memory after a single stimulation. Additionally, the present results showed that only the participants who received the actual stimulation showed reduced brain activation during a memory task. Thus, the present finding further suggested that PNM may affect the hemodynamic response of the brain, which is associated with improving memory.

After the real/sham PNM, the experimental and control groups did not differ in the working memory performance in the *n*-back tasks. However, when the change of working memory-related [oxy-Hb] between the *n*-back tasks in the left, right, and both (overall) prefrontal regions of the brain were compared between the experimental and control groups after real/sham PNM, the experimental group, as compared to the control group, demonstrated a significant reduction in the change of working memory-related [oxy-Hb] as measured by the working memory load index (i.e., the fNIRS response of 3-back condition was subtracted from that of 0-back condition) in the left, right and both prefrontal regions of the brain after the real PNM. These findings suggested that as compared with a less difficult task (i.e., 0-back task), the participant paid less effort in terms of [oxy-Hb] in completing a more difficult task (i.e., 3-back task) after the real PNM. Indeed, the demonstrated significant reduction in the working memory-related [oxy-Hb] after real PNM is consistent with our previous study^25^. Older adults with MCI showed improved visual memory performance and a significant reduction in the [oxy-Hb] after a single PNM session. Consistent with our hypothesis, our results in young healthy adults further demonstrated that participants paid fewer efforts in completing the *n*-back task after receiving a single PNM session but maintained a similar level of behavioral performance at the same time. The visual working memory performance at baseline was also associated with the working memory-related [oxy-Hb] changes after PNM, suggesting that individuals with better memory performance at baseline will benefit more from PNM in reducing the mental efforts. Therefore, PNM may enhance the neural efficiency by reducing the mental efforts necessary for the task with the same level of difficulty, which then makes the task less arduous for the participants. This result was also consistent with the notion that the [oxy-Hb] estimates how effortful an individual is working to accomplish the task^24^.

It is also found that the reaction time was shorter in the control group in the 0-back test. Although the control group also had a faster reaction time in the 3-back test, the group difference was not significant. The shorter reaction time observed in the control group may be due to the random group assignment without matching the *n*-back performance at pre-assessment. However, it is noted that the reaction time difference (i.e., the reaction time of 3-back condition subtracted by that of 0-back condition) was also not significant. Therefore, the significantly faster reaction time in the 0-back test and slightly faster reaction time in the 3-back test probably is not related to group difference in working memory capacities at baseline. Despite this, a future study can consider controlling for all baseline performance during group assignments.

Although the results are encouraging, the current study has a few limitations. Firstly, only young adults with at least an undergraduate qualification were recruited in this study. It is unclear whether the reduced frontal activation phenomenon after PNM will appear in other age or educational cohorts. Future studies should include a sample with diverse ranges of age and education levels. Secondly, this study only examined the effect of a single PNM. The duration of the effect, however, remains unknown. Further studies are needed to understand the best treatment dosage and whether the treatment effects can last for a longer duration after brain stimulation. Lastly, the present study only investigated the hemodynamic responses in the prefrontal cortex. Future studies can consider investigating the hemodynamic responses in other brain regions and changes in a cell and neuronal level to obtain a complete picture of the beneficial effects of PNM on human cognitive function.

In conclusion, this study demonstrated that a single PNM session significantly reduced [oxy-Hb] level during a working memory task, but without reducing the accuracy and the reaction time of the task. This result suggests that this technique may reduce the cognitive effort required to complete a task and contributes to our understanding of the mechanisms of PNM. Future research may consider evaluating the treatment effect in populations with different demographics.

## Methodology

### Participants

Thirty-three participants (16 males), with mean age of 25.24 years (SD = 8.86 years), were recruited through an online advertisement using the university mass email system. The inclusion criteria were: (a) 18–40 years old, (b) no history of head injury or epilepsy, and c) no history of psychological and/or neuropsychological disorders. Additionally, the participants' recent depression and anxiety symptoms were assessed using the Chinese version of the 21-item BDI and BAI. Individuals with scores higher than 30 on the BDI and/or 26 on the BAI, which indicate severe depression or anxiety, respectively, were excluded from this study. All the participants had a normal or corrected-to-normal vision at the time of the experiment. Each participant was paid 100 HKD for their participation.

### Procedure

First, baseline visual and verbal working memory abilities were assessed, demographic information was obtained. This was followed by a 9-min PNM session; *n*-back sessions took place before and after each session. During the *n*-back sessions, participants' task-related hemodynamic responses were recorded using fNIRS. For a PNM session, participants were assigned randomly to either the experimental (*n* = 18) or the control (*n* = 15) group. The experimental group received the real PNM stimulation, while a sham stimulation was applied in the control group. This study was conducted in accordance with the Helsinki Declaration of the World Medical Association Assembly and approved by the Joint Chinese University of Hong Kong‐New Territories East Cluster Clinical Research Ethics Committee on 26 November 2019 (Ref. NTEC-2019-0613). Informed consent was obtained from all individual participants included in the study.

### Baseline assessment

#### Hong Kong list learning test (HKLLT)

The HKLLT was employed to assess the participants' verbal working memory ability. The test comprises an immediate recall, 10-min and 30-min delayed recall, and recognition trials. In the immediate recall trial, participants were given a verbalized list of two-word Chinese characters, repeated 3 times. Afterward, they were asked to recall as many words as they could immediately. After 10 and 30 min, they were asked to recall the same list; however, the experimenter did not repeat the list as before. A recognition test was given immediately after the 30-min recall trial. In this, the participants were given a list of words verbally, and they had to decide which words had appeared in the original list. The total number of words produced in the 3 immediate recall trials, the number of words produced in the 10-min and 30-min recall trials, and the accuracy of the recognition test were used as the primary dependent variable.

#### Rey–Osterrieth complex figure test (Rey–O)

The Rey–O was employed to assess the participants' visual working memory. Similar to the HKLLT, this test comprises copy, immediate recall, 30-min delayed recall, and recognition trials. Participants were first asked to copy a complex figure as accurately as possible and then recall it immediately afterward without any cues, and then again after 30 min. It was followed by the recognition trial, in which they were given 24 figures and were asked to circle those figures that had appeared in the complex figure. The accuracy of the four trials was the primary dependent variable.

### Pre- and post-assessment

#### *n*-back task

An *n*-back task was employed to assess the improvement in working memory after PNM. The present *n*-back task consisted of 0- and 3-back conditions, which required a lower and higher cognitive workload, respectively. During the task, a number of digits were shown on the screen one-by-one for 500 ms, with an inter-trial interval of 500 ms. Participants were required to respond using a mouse according to the *n*-back conditions. In the 0-back condition, participants were asked to press the left (right) button for the zero (non-zero) digit. In the 3-back condition, participants were asked to press the left button if the present digit was the same as the one shown three digits ago. If not, they needed to press the right button.

A block design task was used in which participants underwent two 0-back and two 3-back conditions consecutively, and the order was counterbalanced across participants (i.e., either 0–3–0–3-back or 3–0–3–0-back sequence). Before and after each test block, there was a 30-s resting block during which the participants were asked to sit still and do nothing. Throughout the experiment, participants were told to minimize their movements. Each task block contained 20 trials and a 5-s cue was shown on the screen before the first trial, indicating which condition the participants had to undergo. The accuracy and reaction times were the primary dependent variables.

### fNIRS recording

**Figure 4:**
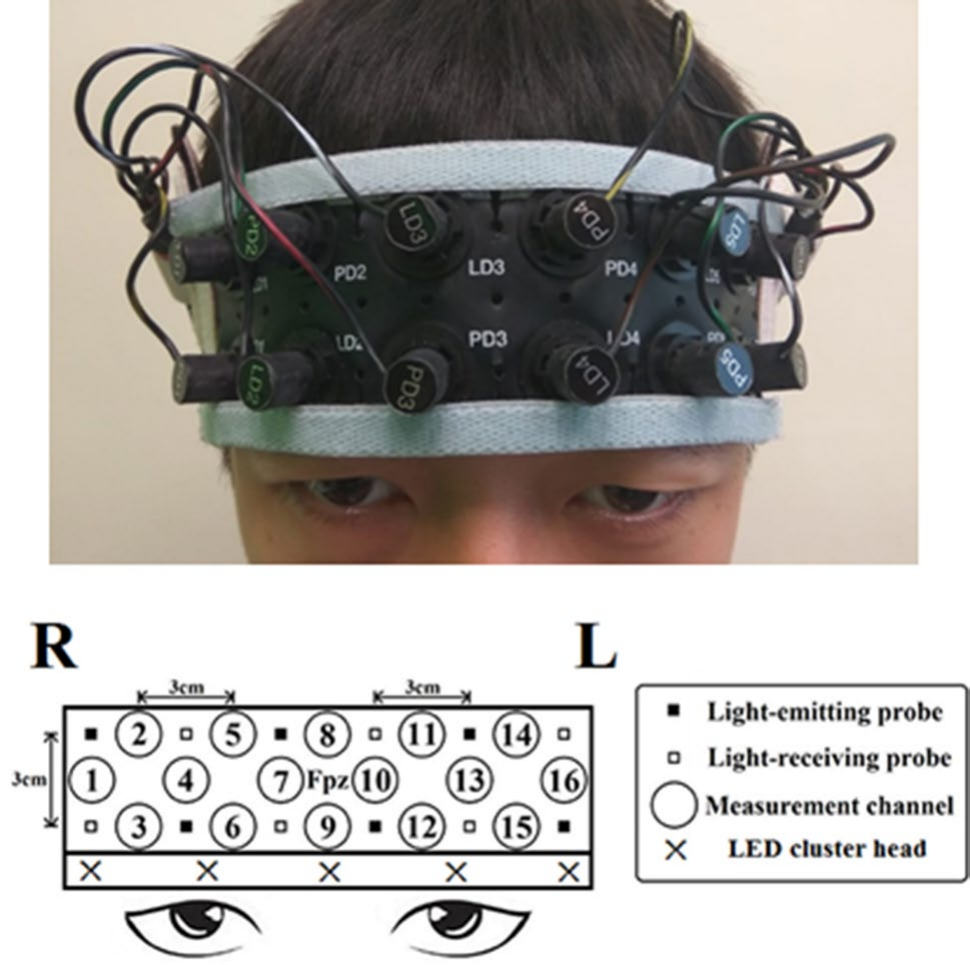
Headset including the PNM and fNIRS devices with 16 probe distribution locations.

The relative [oxy-Hb] and [deoxy-Hb] changes during the *n*-back task were recorded using a 16-channel OEG-SpO2 system (Spectratech Inc., Tokyo, Japan). By utilizing near-infrared light with wavelengths of 770 and 840 nm, the device calculated the participants' task-related hemodynamic responses based on the modified Beer-Lambert Law^30^. Six pairs of emission and detector probes were arranged in a 2 × 6 matrix on each participant's forehead (Fig. 4), with the center of the matrix on Fpz according to the international 10/20 system. The distance between the emission and detector probes was 3 cm, and the sampling rate was 12.21 Hz. The [oxy-Hb] signal was chosen as the primary dependent variable because it demonstrates a stronger relationship with cerebral blood flow compared to the [deoxy-Hb] signal^31^.

### Data analysis of fNIRS

A 0.10 Hz low-pass filter at a slope of 60 dB/octave was applied to remove motion artifacts during the task. Based on the pre- and post-task resting periods, a first-order linear fitting was then performed for each task block to correct the slow drift of signals over time. The pre- and post-task periods were defined as 10 s preceding the task block and the last 10 s of the following resting block, respectively. The corrected fNIRS response was averaged across time points, conditions, and participants. The abovementioned calculations and the data visualization were performed using Matlab R2017a (MathWorks, Natick, MA).

### PNM session

PNM was applied using a model, Wisefor i5-3800 (Wisefor Limited, Hong Kong). This device contained five LED clusters each with a spot area of 1 cm^2^, arranged in a headset, which were distributed evenly across the participant's forehead, and emitted 810 nm continuous wave light at a power density of 20 mW/cm^2^. With a patented design, the temperature of the device was maintained between 36 and 38 °C during the stimulation, and the protocol could be adjusted using a smart phone. This device has obtained a CE certificate for medical devices that have passed the standard of EN 60601-1-2001, 60601-2-2015, 60601-2-57-2011, and was registered at the FDA as a Class 1 device. During the whole PNM session, the deviceswitched on with a beep sound and switched off with a beep sound, and the duration was 350 s (three sections). The energy density was 7 J/cm^2^. During this session, participants were asked to sit comfortably on an office chair with protective goggles. In the control group, participants also wore the PNM headset, and the device was turned on with a beep sound but without the energy supply. Therefore, participants did not identify the group they belonged to.

### Data analysis

Demographic information and baseline measures were compared between the experimental and control groups using independent sample *t*-test and chi-square test. For the *n*-back behavioral data, the sensitivity index A', which considered the hit rate and false alarm rate, was calculated for each *n*-back condition^32^. This index ranged from 0 to 1. The larger the index, the better the discriminatory ability of the participant. The correct reaction time was also averaged from the reaction time of the correct trials after excluding the trials with reaction times below 150 ms and 3 SDs above each participant's mean.

For both the behavioral and fNIRS data during the *n*-back task, two-way ANOVA was used to test the group (i.e. control vs. experimental group) × condition (i.e., 0-back vs. 3-back conditions) interaction. Paired sample *t*-tests were conducted to compare the within-group effect of time (i.e., pre- and post-difference) and condition (i.e., the difference in 0- and 3-back conditions). Furthermore, a working memory load index was calculated to obtain the working memory-related frontal activation by subtracting fNIRS response of 3-back condition from that of 0-back condition. Then, repeated measures ANOVA was also performed to investigate the time (i.e., pre- vs. post-difference) × group (i.e., control vs. experimental group) interaction on such activation. Additionally, Pearson's coefficients were calculated to investigate the correlation between the [oxy-Hb] changes after PNM and other baseline measures. All statistical analyses were performed using SPSS 24.0 software (IBM Corporation, Armonk, NY, USA), with 0.05 significance level.
